# Epigallocatechin-gallate loaded BSA nanoparticles as innovative anti-inflammatory agents in immature macrophages

**DOI:** 10.3389/fbioe.2025.1666492

**Published:** 2025-10-07

**Authors:** Simona Martano, Mariafrancesca Cascione, Livia Giotta, Loris Rizzello, Riccardo Di Corato, Stefano Leporatti, Rosaria Rinaldi, Valeria De Matteis

**Affiliations:** ^1^ Department of Mathematics and Physics “Ennio De Giorgi”, University of Salento, Lecce, Italy; ^2^ Department of Biological and Environmental Sciences and Technologies (DiSTeBA), University of Salento, Lecce, Italy; ^3^ Department of Pharmaceutical Sciences (DISFARM), University of Milan, Milan, Italy; ^4^ Institute for Microelectronics and Microsystems (IMM), Italian National Research Council (CNR), Lecce, Italy; ^5^ Center for Biomolecular Nanotechnologies, Istituto Italiano Di Tecnologia (IIT), Arnesano, Italy; ^6^ CNR Nanotec-Istituto Di Nanotecnologia, Lecce, Italy; ^7^ Department of Experimental Medicine (DiMeS) ‐University of Salento Centro Ecotekne, Lecce, Italy

**Keywords:** BSA NPs, desolvation method, inflammation, epigallocatechin-gallate, antioxidant

## Abstract

**Introduction:**

The development of innovative anti-inflammatory therapies is critical for addressing chronic inflammatory diseases and cancer. Epigallocatechin gallate (EGCG), a polyphenolic compound with strong antioxidant and anti-inflammatory properties, suffers from limited stability and bioavailability. Bovine Serum Albumin Nanoparticles (BSA-NPs), due to their biodegradability, non-toxicity, and high binding capacity, represent a powerful delivery system for bioactive compounds.

**Methods:**

EGCG-loaded BSA nanoparticles (EGCG@BSA-NPs) were synthesized via the desolvation method. The nanoparticles were characterized by Transmission Electron Microscopy (TEM), Dynamic Light Scattering (DLS), ζ-potential analysis, Fourier-transform infrared spectroscopy (FTIR), and UV-Vis spectroscopy. Encapsulation efficiency and antioxidant capacity were assessed by Trolox equivalent antioxidant capacity (TEAC) assays. The anti-inflammatory potential was evaluated in immature macrophages (THP-1 cells) by assessing NF-κB nuclear translocation and the stimulation of proinflammatory cytokines IL-8 and TNF-α.

**Results:**

Morphological and physicochemical analyses confirmed the successful formation of spherical EGCG@BSA-NPs with improved size uniformity and controlled surface charge. Antioxidant assays demonstrated enhanced radical scavenging activity compared with unloaded BSA-NPs and free EGCG. Cellular studies showed that EGCG@BSA-NPs reduced NF-κB nuclear translocation and decreased IL-8/TNF-α secretion, highlighting their anti-inflammatory efficacy.

**Discussion:**

These findings suggest that EGCG@BSA-NPs are an effective nanoplatform for the controlled delivery of polyphenolic compounds. By improving stability and enhancing bioactivity, they hold significant promise in modulating macrophage function and reducing inflammation, thereby supporting their potential use in chronic inflammatory disease and cancer therapy.

## 1 Introduction

Inflammation is a tightly controlled biological process, necessary for both tissue repair and pathogen defense (Chen et al., 2018; Burini et al., 2020). However, persistent or dysregulated inflammation is associated with the pathophysiology of a wide range of chronic diseases, including cancer, cardiovascular disease, neurodegenerative disorders, and autoimmune conditions (Bhol et al., 2024; Burini et al., 2020; Chavda et al., 2023).

As important innate immune system effectors, macrophages are essential for coordinating inflammatory reactions (Duque and Descoteaux, 2014). They can polarize into different functional phenotypes in response to environmental inputs, demonstrating their good adaptability (Chen et al., 2023). While alternatively activated macrophages (M2) aid in tissue repair and inflammation resolution, classically activated macrophages (M1) encourage pro-inflammatory responses by releasing high concentrations of reactive oxygen species (ROS), nitric oxide (NO), and pro-inflammatory cytokines (e.g., TNF-α, IL-1β, and IL-6) (Arora et al., 2018; Wang et al., 2019). One possible treatment approach for reducing inflammatory disorders is to target macrophage polarization (Tsai et al., 2022). There is a great deal of therapeutic interest in methods that lower oxidative stress and decrease M1 polarization while increasing M2 activation. Natural polyphenols with anti-inflammatory, immunomodulatory, and antioxidant qualities, such epigallocatechin gallate (EGCG), have been extensively researched (Singh et al., 2011; Kohli and Alpar, 2004). The most prevalent catechin in green tea, EGCG, has been shown to scavenge free radicals, block the signaling pathways of NF-κB and STAT1, and modify the polarization of macrophages toward an anti-inflammatory M2 phenotype (Mokra et al., 2023). However, because of its instability, poor water solubility, and quick metabolic breakdown, EGCG is still difficult to use in therapeutic settings (Joo et al., 2012; Qureshi et al., 2024). Today the use of nanotecnologies in biomedical applications is rapidly expanding (De Matteis et al., 2016; Asil et al., 2020; Hoyt and Mason, 2008) Nanoparticle-driven delivery systems provide an effective method to address the challenges linked to EGCG administration. By enclosing EGCG in nanocarriers, one can protect the molecule from oxidative deterioration, improve its bioavailability, and facilitate controlled release (Granja et al., 2016; Granja et al., 2017). Specifically, protein-based NPs have attracted considerable interest because of their biocompatibility, biodegradability, and natural binding properties. Among these, bovine serum albumin (BSA) is extensively utilized in nanoparticle fabrication due to its remarkable physicochemical characteristics, such as solubility in water, lack of immunogenic response, and various functional groups ideal for drug conjugation (Solanki et al., 2021; Granja et al., 2017; Spada et al., 2021). BSA-NPs can be produced through gentle desolvation techniques, resulting in stable, nanoscale carriers that can encapsulate various bioactive compounds (Bartlett et al., 2025; Weber et al., 2000).

Desolvation methods represent one of the most widely approach to obtain BSA nanostructures. In this approach, a desolvating agent such as ethanol or acetone is gradually added to an aqueous BSA solution, leading to controlled protein aggregation through reduction of solubility. Subsequent crosslinking or thermal stabilization ensures the formation of monodisperse NPs with tunable size and surface properties (Jahanban-Esfahlan et al., 2016; Tanjung et al., 2024; Hasan and Ghareeb, 2024). This technique is considered particularly advantageous because it does not require harsh conditions, preserves the biological activity of encapsulated compounds (Bartlett et al., 2025; Singh and Srivastav, 2025). In addition, desolvation approach allows for high encapsulation efficiency of sensitive bioactive molecules particularly susceptible to environmental conditions (Kamble et al., 2025).

Crucially, carriers based on albumin can utilize innate biological routes for precise delivery. BSA is recognized for its interaction with gp60 receptors and SPARC proteins, which are prominently expressed in both tumor tissues and inflamed areas, aiding in the passive targeting of affected regions (Ji et al., 2024; Lin et al., 2016). Recent research has effectively shown the application of BSA-NPs for the delivery of anti-inflammatory agents, antioxidants, and immunomodulators, resulting in improved therapeutic results (Zhang et al., 2025; Ifijen et al., 2025). Although considerable advancements have been made, the use of EGCG-loaded BSA-NPs for specifically influencing immature macrophage inflammatory responses is still not well researched (Li et al., 2022). Immature macrophages, noted for their increased plasticity and sensitivity to environmental signals, serve as a prime target for early intervention in inflammatory events (Huang et al., 2024). By delivering EGCG in a protected and bioavailable form, it may redirect macrophage activation toward a pro-resolving, M2-like phenotype, thereby attenuating both the onset and progression of chronic inflammation. (Song et al., 2023). In this work, we presented the design, synthesis, and characterization of BSA-NPs loaded with EGCG (EGCG@BSA-NPs) using a desolvation technique followed by thermal stabilization. We assessed the morphology, size distribution, surface charge, chemical interactions, and antioxidant potential of the NPs. Additionally, we explored the effect of EGCG@BSA-NPs on immature macrophages, i. e., THP-1 cell lines concentrating on their ability to influence inflammatory responses. Our results indicated that EGCG@BSA-NPs acted as a potential nanoplatform for modulating macrophage-driven inflammation, providing new knowledges for anti-inflammatory treatments.

## 2 Materials and methods

### 2.1 Synthesis of BSA-NPs

BSA-NPs were synthesized using a desolvation technique. Specifically, 25 mg of BSA (Sigma-Aldrich, Dorset, United Kingdom), powder was dissolved in 500 μL of 10 mM NaCl solution. Under constant magnetic stirring (400–600 rpm) at room temperature, 4.0 mL of ethanol were added dropwise to the BSA solution. The resulting suspension was subjected to thermal denaturation at 70 °C for 30 min.

### 2.2 Synthesis of EGCG-loaded BSA-NPs (EGCG@BSANPs)

EGCG@BSA-NPs were prepared following the same desolvation protocol. Epigallocatechin gallate (EGCG) powder (Sigma-Aldrich, Dorset, United Kingdom) was dispersed in the 4.0 mL of ethanol used for the desolvation step at final concentrations of either 327 μM (0.6 mg) or 1 mM (1.83 mg), depending on the sample. The detailed synthesis parameters for each sample were listed below, where each formulation varied in EGCG concentration, synthesis temperature, and final denaturation time.

### 2.3 Post-synthesis processing of NPs

After NPs synthesis, all suspensions were centrifuged at 13.400 rpm for 10 min. The supernatants were collected for subsequent analysis. The resulting pellets were washed with a 1:1 (v/v) mixture of ethanol and Milli-Q water to remove unreacted reagents. For each sample, one aliquot of the pellet was freeze-dried at −55 °C, while the remaining material was stored at 4 °C for further use.

### 2.4 Transmission Electron Microscopy (TEM)

TEM analyses were conducted using a JEOL JEM-1011 microscope (TEM, Jeol LTD., Tokyo, Japan) operating at an accelerating voltage of 100 kV. The instrument was equipped with a high-contrast objective lens and a tungsten filament electron source, with an ultimate point resolution of 0.34 nm. Image acquisition was carried out using an 11-megapixel Quemesa CCD camera (Olympus). For TEM sample preparation, approximately 10 µL of the NP suspension was deposited onto carbon-coated copper grids. Following complete evaporation of the solvent, the grids were subjected to microscopic analysis.

### 2.5 Dynamic light scattering (DLS) and ζ-potential

DLS and ζ-potential measurements of BSA-NPs and EGCG@BSA NPs were performed using a Zetasizer Nano-ZS (Malvern Instruments Ltd., Malvern, United Kingdom), equipped with a 4.0 mW HeNe laser operating at 633 nm (ZEN3600). Measurements were carried out in aqueous solution at 25 °C and pH 7. Prior to analysis, nanoparticle suspensions were vortexed for 5 min to ensure homogeneity.

### 2.6 UV-Vis and fourier transform infrared (FTIR) characterization

UV-Vis absorption spectra of the NP suspensions were recorded using a Varian Cary 5,000 spectrophotometer. Infrared spectra were acquired using a FTIR spectrometer (Spectrum One, Perkin Elmer, Waltham, MA, United States) equipped with a universal attenuated total reflectance (ATR) module. The internal reflection element consisted of a three-bounce diamond microprism with a diameter of 4 mm. For ATR-FTIR measurements, approximately 2 µL of the NP aqueous solution was deposited onto the crystal surface, and the solvent was allowed to evaporate prior to spectral acquisition at a resolution of 4 cm^−1^.

### 2.7 Antioxidant capacity assessment

The antioxidant capacity of the samples was evaluated through the well-established ABTS decolorization assay using EGCG as standard. The ABTS decolorization assay assesses the ability of antioxidants to quench the ABTS^•+^ radical cation, a chromophore with characteristic absorbance peaks at 645, 734, and 815 nm in aqueous environment. Antioxidants reduce ABTS^•+^, resulting in a measurable decrease in absorbance, which is directly proportional to the antioxidant concentration.

The ABTS^•+^ radical was generated by mixing 1 mL of ABTS solution in MilliQ water (7 mM) with 1 mL of potassium persulfate (4.9 mM), followed by incubation in the dark at room temperature for 12–16 h. The ABTS^•+^ radical was then diluted in PBS to reach an initial absorbance near 0.7 at 734 nm. Measurements were performed using a Perkin–Elmer Lambda 2 UV/Vis spectrophotometer (Perkin–Elmer, Jügesheim, Germany). For analysis, 50 μL of each sample or standard were mixed with 950 μL of diluted ABTS in Eppendorf tubes, vortexed for 10 min, and then absorbance at 734 nm was measured. Standards included S1 (327 μM EGCG, 0.6 mg) and S2 (1 mM EGCG, 1.83 mg); the blank was PBS, and unloaded BSA-NPs served as control.

### 2.8 Time-dependent oxidative degradation (stability assay)

Pure epigallocatechin gallate (EGCG) was used as a standard. BSA-NPs loaded with EGCG were previously prepared using the desolvation method, quantified, and stored at 4 °C until use. Phosphate-buffered saline (PBS) 1X, pH 7.4, was used as the incubation medium. All samples were filtered using 0.22 µm PTFE syringe filters prior to analysis. Absorbance measurements were performed using a UV-Vis spectrophotometer at 280 nm. Two experimental conditions were analyzed.

• *Free EGCG*: A solution of EGCG in PBS 1X was prepared at a final concentration of 100 μg/mL.
• *Encapsulated EGCG@BSA-NPs:* EGCG@BSA-NPs were suspended in PBS 1X to achieve an equivalent EGCG concentration (100 μg/mL).

Samples were incubated by using 1.5 mL microcentrifuge tubes at 37 °C in the dark (or under reduced light conditions) to simulate extracellular physiological conditions. Aliquots of 200 µL were collected at the following time points: 0, 1, 2, 4, 8, and 24 h. After collection, aliquots were stored at 4 °C until analysis. Prior to quantification, each sample was filtered using a 0.22 µm and absorbance was measured at 280 nm using a quartz cuvette with 1 cm path length.

The percentage of EGCG remaining at each time point was calculated using the formula:

$$\%\;EGCG\;remaining=\left(\frac{A_{t}}{A_{0}}\right)*100$$

was the initial absorbance at time zero. The data were plotted as % EGCG remaining *versus* incubation time to assess the oxidative stability of the free *versus* encapsulated NPs.

### 2.9 THP-1 culture and differentiation

Human leukemic monocytes (THP-1) were purchased from the American Type Culture Collection (ATCC-THP-1 TIB-202 ^™^) and cultured and maintained in RPMI-1640 (Sigma-Aldrich, Dorset, United Kingdom), containing 2 mM l-glutamine and 25 mM Hepes (Sigma-Aldrich, Dorset, United Kingdom), and supplemented with 10% (v/v) heat-inactivated fetal bovine serum (FBS, Sigma-Aldrich, Dorset, United Kingdom), 1% (v/v) penicillin-streptomycin (Sigma-Aldrich, Dorset, United Kingdom), and 0.1% (v/v) amphotericin B (Sigma-Aldrich, Dorset, United Kingdom). The THP-1 cells were used for *in vitro* experiments of passage numbers nine and twenty. The *in vitro* experiments with these cells were carried out between passage numbers three and nine. Prior to the *in vitro* cellular studies, THP-1 cell differentiation into a mature macrophage-like state (M0-macrophages) was induced through incubation with 10 ng/mL of phorbol 12-myristate 13-acetate (PMA, Sigma-Aldrich, Dorset, United Kingdom) for 48 h in a humidified atmosphere, with 95% air and 5% CO_2_, at 37 °C.

### 2.10 Viability assay (MTT)

The THP-1 were seeded at a concentration of 5 × 10^3^ cells per well in 96-well plates and differentiated as mentioned above in a humidified atmosphere, with 95% air and 5% CO_2,_ at 37 °C. Then, three concentrations of BSA NPs (0.5 mg/mL, 1 mg/mL and 2 mg/mL), three concentrations of EGCG free (5 μM, 10 μM and 20 µM) and three concentrations of EGCG@BSA (0.5 mg/mL – 4.91 µM, 1 mg/ml 9.81 and 2 mg/mL– 19.62 µM) for 6 h and 24 h. Control wells were incubated with equivalent volumes of a cell culture medium and/or solution of 10% (v/v) dimethyl sulfoxide (DMSO, Sigma-Aldrich, Dorset, United Kingdom) in DPBS A WST-8 (Sigma-Aldrich, Dorset, United Kingdom) assay was performed following the procedure previously described in (De Matteis et al., 2017). The data were expressed as the mean ± SD.

### 2.11 Reactive oxigen species (ROS) attenuation assay

THP-1 cells were seeded in 96-well microplates and treated with H_2_O_2_ to stimulate the oxidative stress for 3 h. Then, three concentrations of BSA-NPs (0.5 mg/mL, 1 mg/mL and 2 mg/mL), three concentrations of EGCG free (5 μM, 10 μM and 20 µM) and three concentrations of EGCG@BSA (0.5 mg/mL–4.91 μM, 1 mg/mL-9.81 μM and 2 mg/mL – 19.62 μM) were used. After 6 h and 24 h the reduction of ROS was recorded following the cell–NP interaction the DCF-DA (2′,7′-dichlorofluorescein diacetate, Sigma-Aldrich, Dorset, United Kingdom) assay-test (Abcam, Cambridge, United Kingdom) following manufacturer’s instructions. Test was performed onto microplates following the procedure reported in (De Matteis et al., 2021). The H_2_DCFDA intensity was measured with a microplate reader and the fluorescence excitation/emission was 485/535 nm. Fluorescent images were captured using a Zeiss LSM900 Airyscan confocal laser scanning microscope with Zen 3.2 software (Carl Zeiss, Jena, Germany). Data were expressed as mean ± SD. Differences in ROS generation between cells treated with NPs and controls were considered statistically significant with a *p*-value < *0.05.

### 2.12 Cytokines responses

The release of cytokines IL-8 and the growth factor, TNF-α, was quantified by an enzyme-linked immunosorbent assay (ELISA) on THP-1 (after differentiation) exposed to three concentrations of BSA-NPs (0.5 mg/mL, 1 mg/mL and 2 mg/mL), three concentrations of EGCG free (5 μM, 10 μM and 20 µM) and three concentrations of EGCG@BSA-NPs (0.5 mg/mL – 4.91 µM, 1 mg/mL 9.81 and 2 mg/mL – 19.62 µM). After the centrifugation step (2000 × g for 10 min), the supernatants from the cultures containing 0.5× 10^6^ cells/mL in a final volume of 1 mL were collected and stored at −80 °C until the analyses. Human IL-8 and TNF-α ELISA kits (Abcam, Cambridge, United Kingdom) were used, following the manufacturing procedure, after the calibration curve construction. The reactions were quantified by spectrophotometry.

### 2.13 NF-κb signaling imaging and quantification assay

NF-κB signalling imaging was performed using a Confocal Laser Scanning Microscope (CLSM, Leica SP8, Milton Keynes, United Kingdom). Firstly, the THP-1 cells were seeded at a concentration of 5 × 104 cells per glass-bottom Petri dish (Ibidi) and differentiated as reported in (De Matteis et al., 2021). Then, H_2_O_2_, BSA- NPs, free ECGC (10 µM) and EGCG@BSA-NPs (1 mg/mL of BSA with 9.81 µM ECGC internalized) for 24 h in humidified atmosphere, with 95% air and 5% CO_2_, at 37 °C. Following the treatment, the cells were washed with DPBS and fixed using 3.7% formaldehyde (Sigma-Aldrich, Dorset, United Kingdom) for 10 min at room temperature. After the fixation step, followed by DPBS washing for the membrane permeabilization step, the cells were incubated with 0.2% Triton-X (Sigma-Aldrich, Dorset, United Kingdom) for a further 10 min at RT. Then, immunostaining blocking was performed, using 5% of BSA (Sigma-Aldrich, Dorset, United Kingdom) to prevent unspecific antibody binding. After 1 h at RT, the cells were incubated with NF-κB p65 Antibody (F-6) and FITC (Santa Cruz Biotechnology Inc., Heidelberg, Germany) diluted in 1% BSA overnight in a humidified chamber at 4 °C. After 24 h, the cells were washed with DPBS, and the nuclei were stained with Hoescht 33342 (Sigma-Aldrich, Dorset, United Kingdom) for 10 min at RT. At least 10 different regions of the petri dishes were acquired by a confocal microscope, and an NF-κB nuclear translocation imaging analysis was evaluated by co-localization (Pierce’s coefficient values) of the NF-κB and nucleus fluorescence intensity signals using the Fiji ImageJ software (version 2.0). The plasma membrane was stained using a CellMask™ Deep Red Plasma Membrane Stain (Thermo Fisher Scientific, Waltham, MA, United States), and nuclei were stained with Hoechst 33342 (Thermo Fisher Scientific, Waltham, MA, United States).

### 2.14 Statistical analysis

Statistical analyses were performed using OriginPro (version 8.1). The difference between three and more groups was analyzed through one-way or two-way ANOVA multiple comparisons, respectively. The differences between two groups were evaluated by a two-tailed Student’s-test. The differences were statistically significant when *p < 0.05.

## 3 Results and discussion

The development of a stable and biocompatible nanocarrier is essential for the effective delivery of sensitive polyphenolic compounds like EGCG, whose therapeutic potential is often limited by rapid degradation and low solubility (Yang et al., 2020). In this context, BSA-NPs represent an ideal platform due to their biocompatibility, biodegradability, and innate ability to bind and protect biomolecules (Stringer et al., 2017). By leveraging the structural and functional properties of albumin, EGCG@BSA-NPs offer a dual advantage: they stabilize the antioxidant compound and enable its controlled release in biological environments. The following results demonstrated how this nanocarrier system successfully preserves EGCG activity, enhances its antioxidant capacity, interacting with immune cells. These findings underscore the relevance of albumin NPs not only as passive carriers but also as bioactive delivery tools capable of modulating inflammation at cellular level.

**FIGURE 1:**
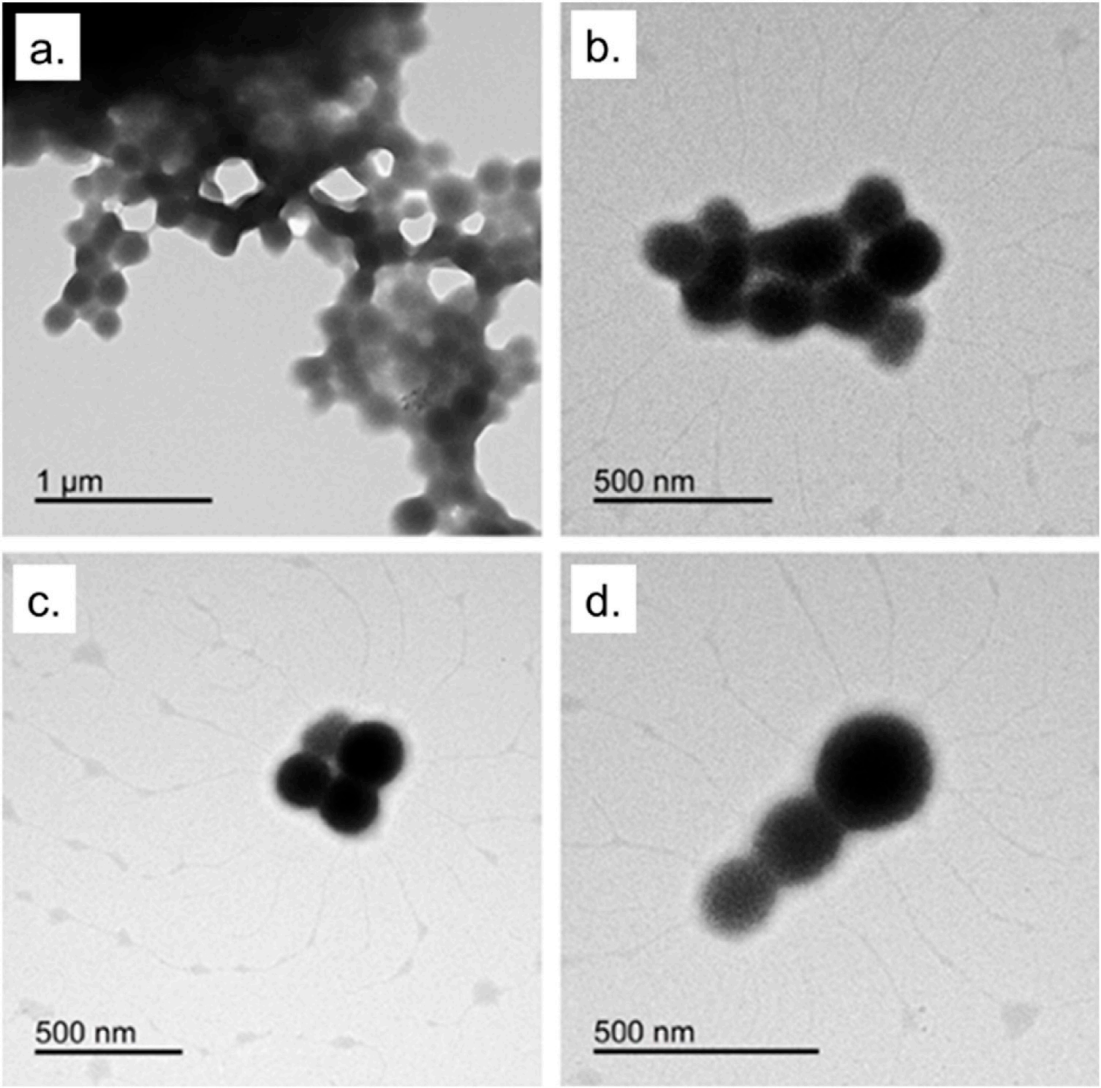
TEM images of BSA-NPs from desolvation method described in the materials section.

Indeed, recent evidence highlights that BSA-NPs themselves can exert a mild immunomodulatory role, influencing cytokine secretion and ROS generation in macrophages (Liu et al., 2022). This suggests a synergistic contribution when bioactive compounds such as EGCG are encapsulated, resulting in enhanced antioxidant and anti-inflammatory response. With the aim to evaluate the potential of EGCG@BSA-NPs as anti-inflammatory nanocarriers, we first optimized and characterized their physicochemical properties. The desolvation method successfully yielded well-dispersed, spherical NPs with controlled size and surface charge, as confirmed by TEM (Figure 1).

This synthetic procedure was adopted for protein-based nanosystems because it avoids harsh chemical conditions preserving protein functionality, and maintains the stability of sensitive bioactives (Ahire et al., 2025; Varanko et al., 2020). The obtained NPs displayed a generally spherical morphology with some degree of aggregation, particularly evident in the top panels. In the lower panels, more dispersed particles can be observed, offering a clearer view of their uniform shape. These acquisitions highlight the successful formation of well-defined BSA-NPs, confirming the reliability and efficiency of the desolvation approach used for their synthesis. The observed partial aggregation is a common feature in protein-based NPs, especially under aqueous conditions, as previously reported for BSA and casein nanocarriers (Zhao et al., 2020). Nevertheless, such aggregation does not necessarily compromise biological performance and can sometimes improve uptake through enhanced multivalent interactions with cell membranes (Bélteky et al., 2021).

**FIGURE 2:**
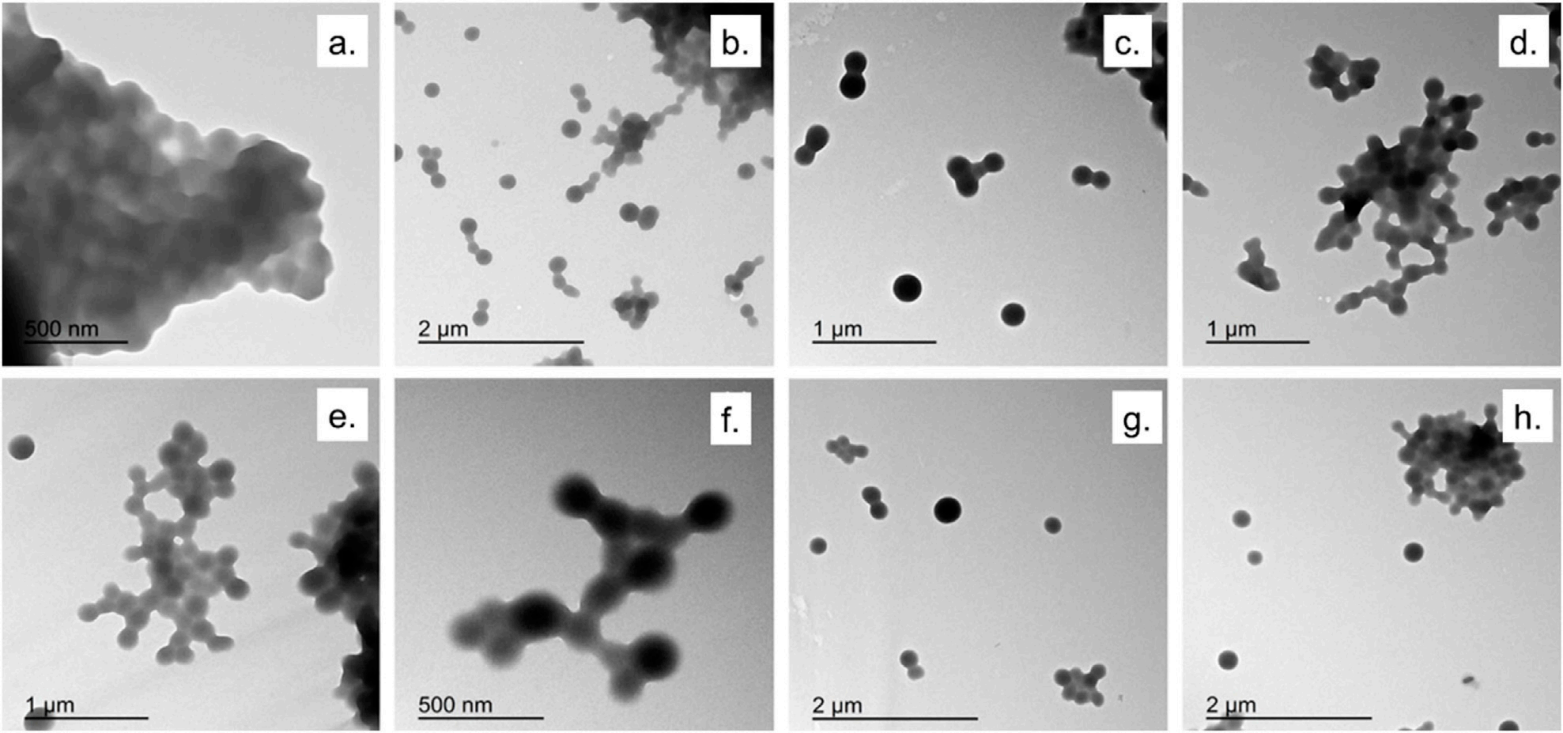
TEM micrographs of EGCG@BSA-NPs at different magnifications. **(a).** NPs synthesized using ECGC 1 mM at room temperature (30 min); **(b–d).** NPs synthesized using ECGC 327 μM at 37 °C (30 min); **(e,f)** NPs synthesized using ECGC 327 μM at room temperature (45 min); **(g,h)** NPs synthesized using ECGC 1 mM at 37 °C (30 min).

**FIGURE 3:**
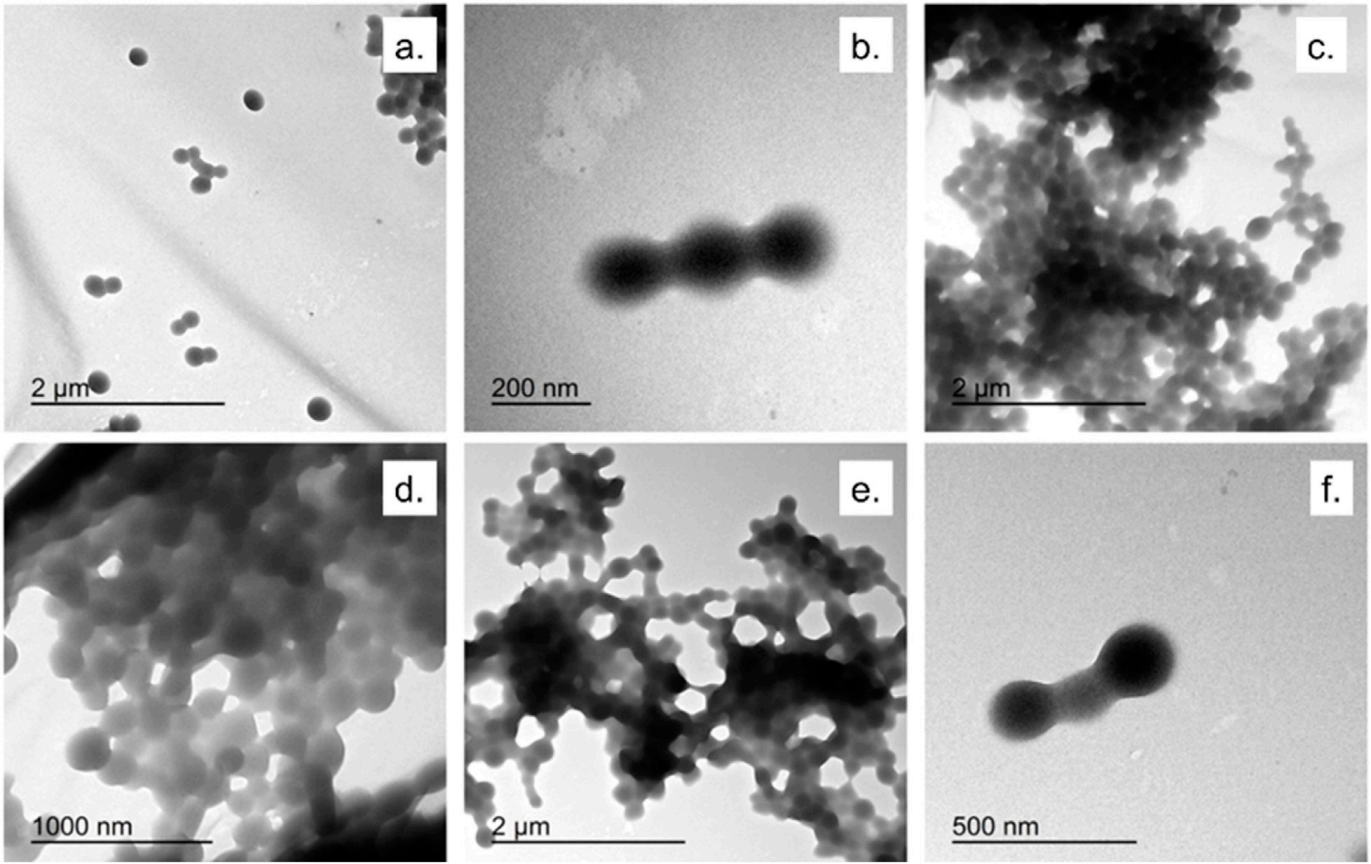
TEM micrographs of EGCG@BSA-NPs at different magnifications. **(a,b)** NPs synthesized using ECGC 1 mM at room temperature (45 min); **(c,d)** ECGC 327 μM at 37 °C (45 min); **(e,f)** NPs synthesized using ECGC 1 mM at 37 °C (45 min).

Figures 2, 3 showed TEM micrographs of EGCG@BSA-NPs, confirming their predominantly spherical morphology. Individual particles were generally well-defined, with diameters estimated between 30 and 100 nm. A notable contrast in dispersion was observed among the samples: while samples b, c, d (Figure 2), showed uniformly distributed NPs with minimal aggregation, other formulations, particularly samples a (Figure 2) and c, d, e, f (Figure 3) revealed marked clustering. This heterogeneity in aggregation behavior could reflected differences in formulation stability, potentially arising from variations in EGCG encapsulation efficiency, surface charge properties, or synthesis parameters (Table 1). The presence of large NPs clusters in samples depicted in Figure 3 further supported the hypothesis of enhanced interparticle interactions under certain conditions.

**TABLE 1:** Experimental conditions adopted for EGCG@BSA-NPs synthesis.

Sample	EGCG concentration	Synthesis temperature	Denaturation time
1	1 mM	Room Temperature	30 min
2	327 µM	37 °C	30 min
3	327 µM	Room Temperature	45 min
4	1 mM	37 °C	30 min
5	1 mM	Room Temperature	45 min
6	327 µM	37 °C	45 min
7	1 mM	37 °C	45 min

For the subsequent experiments, we chose protocol 2 because the NPs proved to be more monodisperse and spherical. DLS measurements conducted on BSA-NPs and EGCG@BSA-NPs were summarized in Table 2. The unloaded BSA-NPs demonstrated a zeta potential of (−29 ± 5) mV, indicative of pronounced electrostatic repulsion and, consequently, good colloidal stability. However, the high polydispersity index (PDI = 0.6) reflects a broad size distribution, suggesting a heterogeneous population likely influenced by partial aggregation or inefficient size control during synthesis. The mean hydrodynamic diameter of (893 ± 35) nm further supported the formation of relatively large nanoparticulate assemblies, consistent with BSA-NPs behavior to aggregate in aqueous environments.

**TABLE 2:** ζ-potential, DLS measurements and PDI values of BSA-NPs and EGCG@BSA-NPs.

Sample	ζ-Potential (mV)	Size (nm)	PDI
BSA-NPs	−29 ± 5	893 ± 35	0,6
EGCG@BSA-NPs	−4 ± 1	700 ± 24	0,3

Upon encapsulation of EGCG, significant alterations in physicochemical properties were observed. The mean particle size decreased to (700 ± 24) nm, accompanied by a reduction in PDI to 0.3, indicating improved size uniformity and potential structural rearrangement induced by EGCG. These findings suggested that EGCG may promote the formation of more compact and homogenous NP structures, possibly by stabilizing the protein matrix and reducing interparticle aggregation. This size reduction is in line with previous works, where hydrogen bonding and hydrophobic interactions between EGCG and BSA drive compaction of the protein matrix.

Notably, the NPs loaded by EGCG presented a surface charge shift from (−29 ± 5) mV to (−4 ± 1) mV (Table 2). This substantial decrease in negative surface potential was likely due to surface charge masking, attributed to EGCG interactions with functional groups on the BSA-NPs surface. These interactions may involve hydrogen bonding, hydrophobic association, or electrostatic complexation. Although indicative of successful EGCG incorporation, the reduced zeta potential could compromise colloidal stability over time due to diminished electrostatic repulsion.

**FIGURE 4:**
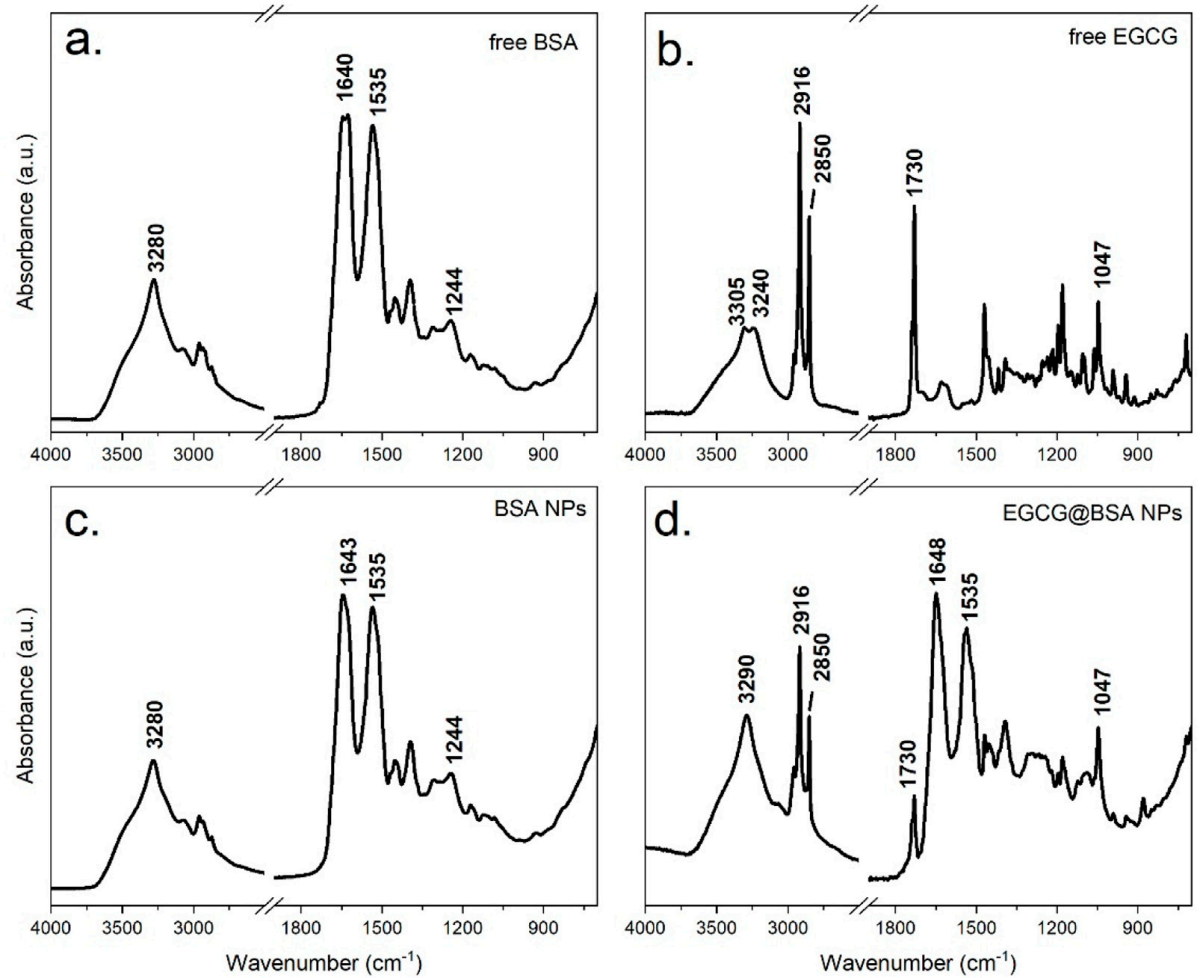
ATR-FTIR spectra of dry films of the following samples: Free BSA **(a)**, BSA-NPs **(b)** Free EGCG **(c)** and EGCG@BSA-NPs **(d)**.

FTIR analysis was conducted to investigate molecular interactions during NPs synthesis, with particular attention to the influence of sodium chloride (NaCl) and the incorporation of EGCG in NPs core. As shown in Figure 4, NaCl employed in the desolvation process, did not contribute to infrared absorption bands due to its lack of a changing dipole moment strong enough to interact with mid-infrared light. Although spectroscopically silent, NaCl may exerted a minor physical effect by crystallizing on the ATR diamond surface, potentially hindering NP adhesion. Nonetheless, the amphiphilic nature of BSA enables the formation of a stable interfacial layer, promoting strong interaction with the ATR crystal. This interfacial stability likely compensated for any potential interference introduced by salt crystallization.

To elucidate the specific interactions between EGCG and the protein matrix, a comparative FTIR analysis was performed on four distinct samples: native BSA (unmodified protein), BSA-NPs, pure EGCG and EGCG@BSA-NPs. The resulting spectra, presented in Figure 4, provided insight into structural changes upon NP formation and compound encapsulation.

The FTIR spectrum of native BSA (Figure 4a) showed well-defined bands characteristic of its peptide-based structure. A broad absorption around 3,300 cm^−1^ (Amide A) corresponded to O–H and N–H stretching vibrations involved in hydrogen bonding. Prominent features of the protein backbone included the Amide I band (∼1,640 cm^−1^), primarily arising from C=O stretching of peptide bonds, and the Amide II band (∼1,535 cm^−1^), attributed to N–H bending and C–N stretching. Additional features included the Amide III band (∼1,240 cm^−1^), linked to protein folding, and C–H stretching vibrations near 2,900 cm^−1^, associated with aliphatic side chains.

The FTIR spectrum of pure EGCG (Figure 4b) was consistent with its polyphenolic nature. A broad O–H stretching band appears between 3,200 and 3,500 cm^−1^, while ester C=O groups absorb near 1730 cm^−1^ producing a characteristic split band. Aromatic C=C stretching bands (1,500–1,600 cm^−1^) and C–O stretching (1,000–1,300 cm^−1^) further confirm EGCG’s identity.

Upon NP formation, the BSA-NPs spectrum (Figure 4c) retained the core protein fingerprint, but notable changes occur. The disappearance of ethanol-related signals confirms effective washing and solvent removal. Amide I and II bands remain evident (∼1,645 cm^−1^ and ∼1,535 cm^−1^, respectively), although minor shifts and intensity variations suggest structural rearrangement. A broader Amide A band, slightly shifted, points to enhanced hydrogen bonding. Increased absorbance around 1700 cm^−1^ may reflect crosslinking or new interactions stabilizing the nanoparticle matrix. Moreover, intensified absorption between 1,000 and 1,500 cm^−1^ indicated possible chemical modifications introduced during synthesis process.

Following EGCG encapsulation, the FTIR profile of EGCG@BSA-NPs (Figure 4d) exhibited the absorption bands of both components, whose relative intensity highlighted the high EGCG loading onto the BSA-NPs. A change of the intensity ratio of the two components of the split EGCG ester signal was observed following encapsulation indicating specific interactions with the BSA structure.

Overall, the ATR-FTIR spectra confirmed successful NP formation, structural rearrangement of BSA upon desolvation, and effective incorporation of EGCG into the protein network. The observed band shifts align with previously reported protein–polyphenol interactions and support the proposed NP-based delivery mechanism.

The ABTS decolorization assay was employed to quantitatively assess the loading of EGCG antioxidant equivalents onto BSA-NPs. A key parameter obtained from this analysis is the amount of active EGCG encapsulated in BSA-NPs, expressed in antioxidant microequivalents (µeq) per gram of BSA, which facilitates a comparison of encapsulation efficiency and EGCG loading in the various NPs formulations. The complete dataset is presented in Table 3.

**TABLE 3:** Encapsulation efficiency and loading data relevant to EGCG@BSA-NPs based on antioxidant capacity assessment by ABTS assay.

Sample	Applied EGCG (μeq)	Encapsulated EGCG (μeq)	Encapsulation efficiency (%)	EGCG/BSA (μeq/g)
1	4.500	0.266	5.9	10.7
2	1.472	0.234	15.9	9.4
3	1.472	0.245	16.7	9.8
4	4.500	0.268	6.0	10.8
5	4.500	0.271	6.01	10.9
6	1.472	0.219	14.9	8.8
7	1.472	0.180	12.2	7.2

Among all samples, sample 1 exhibited the highest EGCG content, with 10.7 µeq/g BSA, suggesting that its synthesis conditions were particularly favorable for antioxidant loading. Samples 2, 3, 4 and 5 demonstrated consistent values within the 9.4–10.9 µeq/g BSA range, reflecting good reproducibility. Sample 6 presented a moderate decrease (8.8 µeq/g BSA), while Sample 7 displayed the lowest EGCG loading (7.2 µeq/g BSA), indicative of suboptimal encapsulation conditions.

Encapsulation efficiency (%) was also calculated as the ratio between the amount of EGCG incorporated into the NPs and the total amount introduced during synthesis. Interestingly, while Sample 6 showed a moderate loading level (8.8 µeq/g BSA), its yield reached 14.9%, outperforming Samples 5 and 4, despite their comparable or higher EGCG loading. This result suggests improved encapsulation efficiency in Sample 6, likely due to more favorable synthesis parameters. Notably, among formulations with an initial EGCG amount of 4.5 µeq (Samples 1, 4, 5, and 6), only Sample 6 achieved a yield exceeding 14%, whereas the others remained in the range of 5%–6%. This highlights that higher initial EGCG amount does not necessarily translate to improved yield and may instead increase losses during synthesis or post-processing.

Encapsulation metrics were derived using the TEAC-based quantification of EGCG retained in the NPs and the corresponding initial mass values. The EGCG concentration was calculated through the calibration curve. The final microeq/g BSA values were obtained by dividing the amount of EGCG entrapped by the BSA mass present in each formulation.

Samples with an initial EGCG value of 1.472 µeq (Samples 2, 3, 6, and 7) generally yielded higher encapsulation efficiencies (12%–16%) than those with higher initial EGCG (4.5 µeq), which showed consistently lower yields (∼5–6%). These findings imply that reducing the initial antioxidant concentration may enhance encapsulation efficiency. For instance, Sample 3 achieved the highest overall yield (16.7%), indicating particularly effective loading conditions.

The comparison between Samples 1 and 4, both synthesized with an initial EGCG amount of 4.5 µeq, revealed similar yields (∼5%), further suggesting that increasing the starting concentration alone does not improve EGCG incorporation and may instead contribute to greater losses. Factors such as EGCG degradation, retention in the supernatant, or inefficient encapsulation likely contribute to these losses. Ethanol evaporation during synthesis or heating may also have altered EGCG concentration, influencing loading efficiency.

In summary, the variations observed in both EGCG content and yield reflected differences in experimental parameters, including antioxidant concentration, synthesis temperature, and thermal denaturation time. These results underscore the importance of carefully optimizing formulation conditions to maximize both the amount of EGCG incorporated into the BSA-NPs and the overall process efficiency.

**FIGURE 5:**
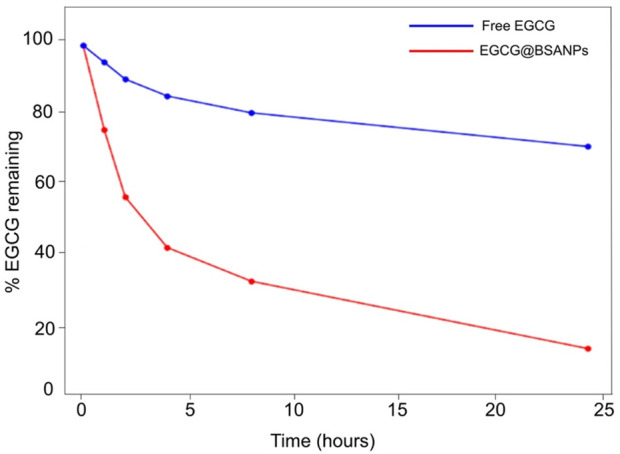
Time-dependent oxidative degradation of EGCG in free form and encapsulated in BSA-NPs. Samples were incubated at 37 °C in PBS (pH 7.4), and the percentage of EGCG remaining was determined at various time points by UV-Vis spectroscopy. Free EGCG (red line) exhibited rapid degradation, while EGCG@BSA-NPs (blue line) showed significantly enhanced stability over 24 h.

To assess the oxidative stability of EGCG when encapsulated within BSA-NPs, a time-dependent degradation study was conducted. Both free EGCG and EGCG@BSA-NPs formulations were incubated at 37 °C in phosphate-buffered saline (PBS, pH 7.4), and the percentage of EGCG retained was quantified at fixed time points using UV-Vis spectroscopy (Figure 5). These conditions were chosen for their suitability to apply them in cells as potent anti-inflammatory tool.

Free EGCG (red line) exhibited a rapid decrease in stability, with approximately 60% degradation occurring within the first 4 h of incubation. By the 24-h time point, only about 10%–15% of the initial EGCG content was retained, indicating a significant oxidative degradation under physiological conditions. In contrast, the EGCG-loaded BSA-NPs (blue line) demonstrated a remarkable enhanced stability. The percentage of encapsulated EGCG remained above 70% at 4 h, gradually decreasing to approximately 60% after 24 h. This sustained retention of EGCG suggests that the BSA matrix effectively shields EGCG from oxidative degradation, likely due to steric protection and reduced exposure to aqueous and oxidative environments.

These evidence confirmed that nanoencapsulation within BSA provided a protective microenvironment for EGCG, significantly improving its chemical stability *in vitro*. This improved stability was particularly advantageous for therapeutic and pharmaceutical applications, where prolonged bioactivity and shelf-life of EGCG were critical. Moreover, these findings supported the potential of BSA-NPs as a promising delivery system for labile polyphenolic compounds such as EGCG.

The nuclear factor kappa-light-chain-enhancer of activated B cells (NF-κB) pathway plays a central role in regulating inflammatory responses in immune cells, particularly macrophages (Gilmore, 1999). Upon exposure to oxidative or pro-inflammatory stimuli, NF-κB is activated and translocate from the cytoplasm to the nucleus, where it promotes the transcription of genes involved in inflammation, immune response, and cell survival (Guo et al., 2024). Targeting NF-κB activation is a well-established strategy for evaluating anti-inflammatory efficacy, and nanoparticle-mediated delivery of polyphenols has been shown to improve cellular uptake and sustained inhibition of this pathway (Guo et al., 2024). Given its pivotal role, NF-κB is widely recognized as a key biomarker for assessing the inflammatory status of macrophages and the efficacy of anti-inflammatory agents. In this context, we investigated the ability of EGCG, both in its free form and encapsulated within BSA-based NP, to modulate NF-κB activation in stimulated macrophages through confocal microscopy and co-localization analysis.

**FIGURE 6:**
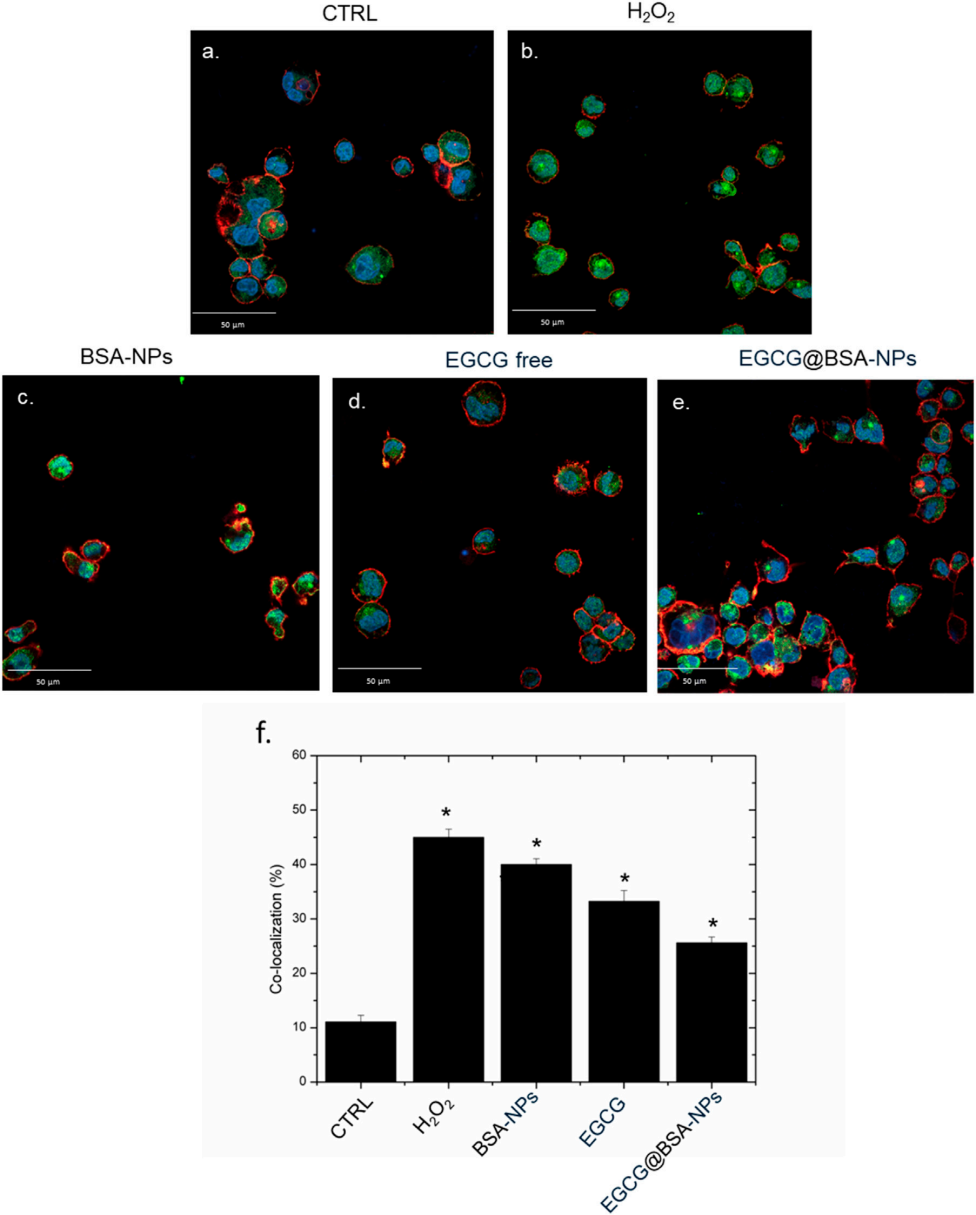
Representative confocal images of untreated macrophages, THP-1 **(a)** exposed to H_2_O_2_, BSA-NPs **(b)**, BSA NPs **(c)** ECGC free (10 M) **(d)** and EGCG@BSA-NPs (1 mg/mL of BSA with 9.81 µM ECGC internalized for 24 h. The cells were fixed and then labeled. The nuclei were stained with Hoechst (blue), Actin cytoskeleton with CellMask™ Deep Red (red), and NF-κB with NF-κB p65 Antibody (F-6) FITC (green intensity signal). Scale bars ware 50 µm. Co-localization analysis (f) of the merged fluorescence signals due to the NF-κB translocation from the cytoplasm to the nucleus (merged blue/green fluorescence intensity signal). The data are expressed as the mean SD (5 images for n = 2) and they were considered statistically significant for *p < 0.05.

Confocal imaging revealed distinct patterns of NF-κB localization in macrophages subjected to various treatments. In untreated control cells (Figure 6a), NF-κB (green) remained predominantly in the cytoplasm, as evidenced by low merge with nuclear Hoechst staining (blue). In contrast, cells exposed to H_2_O_2_ or BSA-NPs (Figures 6b,c) showed pronounced NF-κB nuclear translocation, confirmed by strong co-localization of green and blue signals, thus reflecting the hallmarks of pro-inflammatory triggering.

Treatment conducted with free EGCG (10 µM) led to a modest reduction in nuclear NF-κB signal, suggesting a limited anti-inflammatory activity (Figure 6d). Notably, EGCG@BSA-NPs (1 mg/mL BSA encapsulating 9.81 µM EGCG) Figure 6e significantly reduced NF-κB translocation, with fluorescence distribution closely resembling the control condition. This enhanced efficacy likely results from improved cellular internalization and gradual EGCG release, ensuring sustained modulation of NF-κB activity over time. These observations were quantitatively confirmed by co-localization analysis (Figure 6f), demonstrating a statistically significant decrease in nuclear NF-κB intensity in the EGCG@BSA-NPs group compared to both H_2_O_2_ and BSA-NPs groups (*p < 0.05). These results suggest an enhanced efficacy of EGCG when delivered via NP encapsulation, possibly due to improved cellular uptake and sustained bioactivity, thereby indicating its potential role as a nano-enabled anti-inflammatory approach.

**FIGURE 7:**
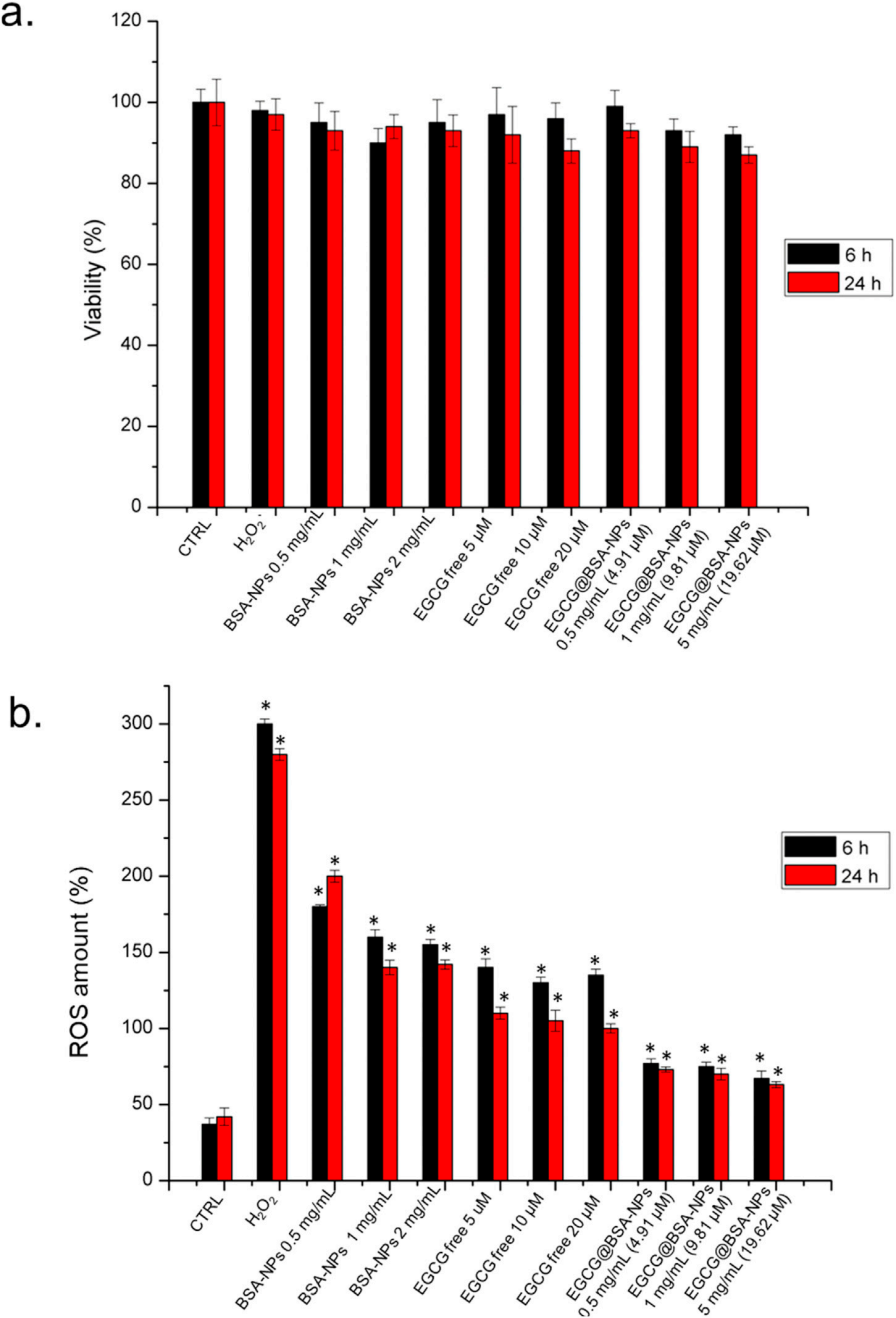
**a)** Viability assay of THP-1 cell lines after 6 h and 24 h of three concentrations of BSA-NPs (0.5 mg/mL, 1 mg/mL and 2 mg/mL), three concentrations of EGCG free (5 μM, 10 μM and 20 µM) and three concentrations of EGCG@BSA-NPs (0.5 mg/mL – 4.91 µM, 1 mg/mL 9.81 and 2 mg/mL – 19.62 µM) The viability of treated cells was normalized to non-treated control cells. As a positive control (P), the cells were incubated with 5% DMSO (data not shown). **(b)** The effect of BSA-NPs, ECGC free and EGCG@BSA-NPs at the concentrations tested for viability assay on the ROS level attenuation after 6 h and 24 h following the procedure described in the section materials was reported. The ROS generation of NP-treated cells is expressed relative to non-treated control cells. As a positive control (P), the cells were incubated with 500 μM of H_2_O_2_. The data are reported as the mean ± SD from three independent experiments; *p < 0.05, compared with the control (n = 8).

Cell viability and intracellular ROS levels were assessed in THP-1 cells following 6 h and 24 h exposure to BSA-NPs, free EGCG, and EGCG@BSA-NPs at three different concentrations. As shown in Figure 7a, cell viability remained above 85% across all treatments and time points, revealing no significant cytotoxic effects from any of the tested formulations. Negligible reductions in viability were observed with increasing concentrations of BSA-NPs and EGCG, both in free and encapsulated formulations, with no substantial difference between two time-points exposures. In contrast, ROS quantification (Figure 7b) revealed a marked and time-dependent depletion of intracellular ROS levels upon treatment. Cells exposed to H_2_O_2_ (positive control) displayed significantly enhanced ROS production, exceeding 250% relative to control levels. Treatment with BSA-NPs and free EGCG led to moderate reductions in ROS, which were further improved in cells treated with EGCG@BSA-NPs. The latter at 1 and 2 mg/mL (corresponding to 9.81 and 19.62 µM EGCG, respectively) achieved the most pronounced ROS suppression, with levels approaching or below baseline by 24 h. These effects were statistically significant compared to untreated controls, thus suggesting that EGCG delivery via BSA-NPs enhances antioxidant efficacy in a dose- and time-dependent manner while maintaining high cell viability.

**FIGURE 8:**
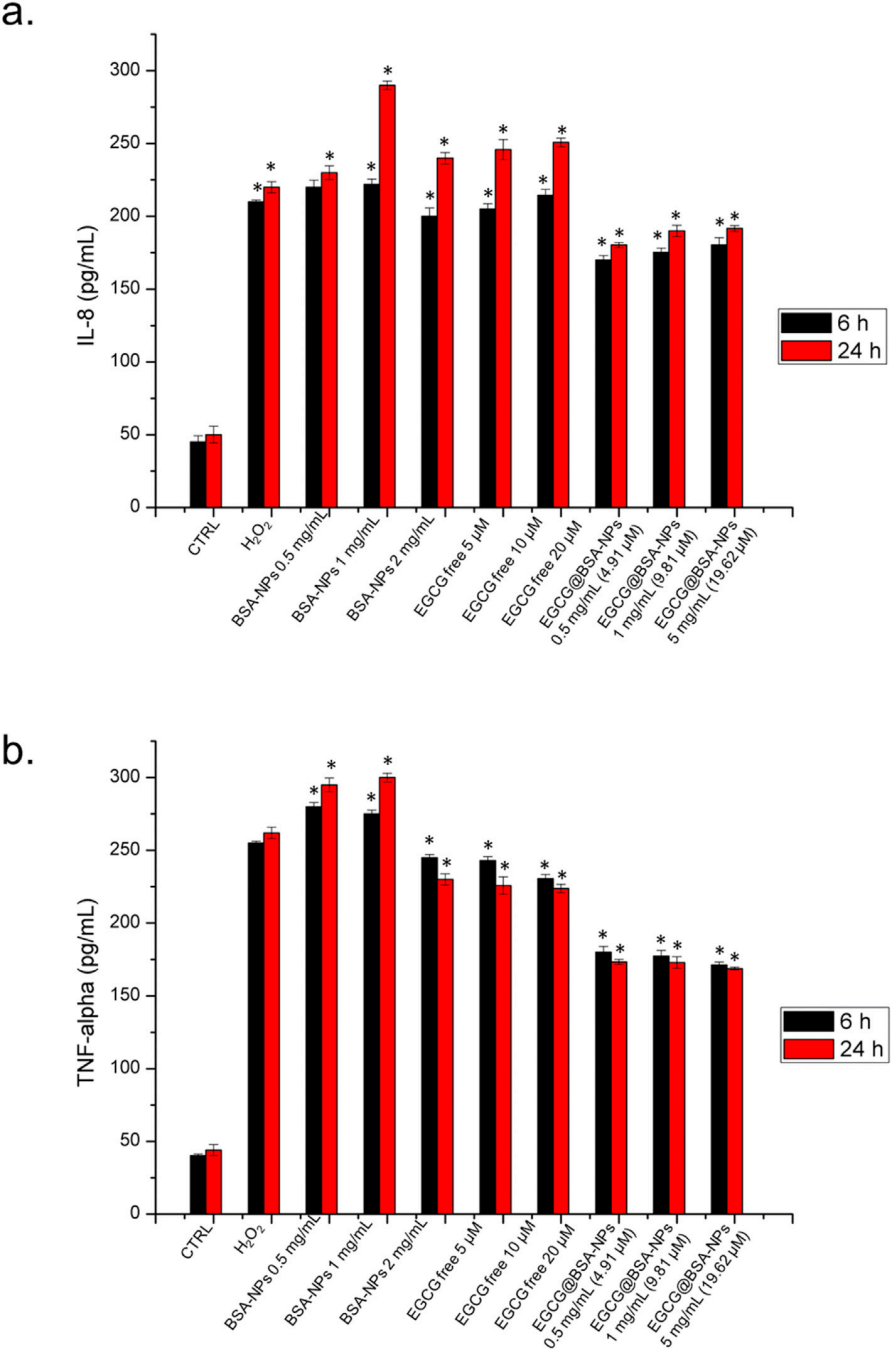
Levels of IL-8 **(a)**, and TNF-α **(b)** in THP-1 cells, expressed as pg/mL. The assay was conducted by incubating cells with three concentrations of BSA-NPs (0.5 mg/mL, 1 mg/mL and 2 mg/mL), three concentrations of EGCG free (5 μM, 10 μM and 20 µM) and three concentrations of EGCG@BSA-NPs (0.5 mg/mL–4.91 µM, 1 mg/mL 9.81 and 2 mg/mL–19.62 µM) for 6 h and 24 h. The cytokine levels were detected in supernatants from the control cells and the treated cells by an ELISA assay. The results were expressed as the mean ± standard deviation of three separate experiments. Data were statistically significant for *p < 0.05 respect to the control of each time point.

The release of pro-inflammatory cytokines such as interleukin-8 (IL-8) and tumor necrosis factor-alpha (TNF-α) is a hallmark of macrophage activation in response to oxidative and immunogenic stimuli. These mediators play crucial roles in amplifying the inflammatory cascade and recruiting immune cells to sites of injury or infection. Assessing their secretion levels provides valuable insights into the inflammatory status of the cellular environment and the potential efficacy of anti-inflammatory compounds. We then examined how free EGCG and EGCG@BSA-NPs modulate IL-8 and TNF-α production in THP-1-derived macrophages, with the aim of evaluating the anti-inflammatory potential of the nanocarrier system compared to the free bioactive molecule. As reported in Figure 8a, IL-8 secretion was significantly increased in cells treated with BSA NPs, particularly at 1 and 2 mg/mL, suggesting a pro-inflammatory response. Free EGCG at 5, 10, and 20 µM resulted in only a partial reduction of IL-8 levels compared to the control, revealing a slight anti-inflammatory effect. IL-8 production was reduced with EGCG@BSA-NPs in a concentration- and time-dependent manner, with the most pronounced decrease observed at 2 mg/mL (19.62 µM EGCG) after 24 h, bringing cytokine levels close to baseline. A similar trend was observed for TNF-α levels, in Figure 8b. BSA-NPs induced a significant increase in cytokine release, while free EGCG promoted a moderate effect. EGCG@BSA-NPs again demonstrated a substantial decrease in TNF-α expression, especially at higher concentrations and longer exposures. All reductions achieved with EGCG@BSA-NPs NPs were statistically significant when compared to untreated control cells at the respective time points. These findings confirmed that EGCG encapsulated in BSA-NPs enhances anti-inflammatory activity more effectively than its free form, as reflected by the suppression of IL-8 and TNF-α secretion.

## 4 Conclusion

In this work, we developed and characterized EGCG@BSA-NPs as a bioengineered nanosystem for targeted inflammation modulation. The desolvation-based synthesis yielded highly monodisperse, spherical NPs with optimized size distribution and surface charge. FTIR and UV-Vis spectroscopy validated the molecular interactions between EGCG and BSA, indicating successful encapsulation without compromising the structural integrity of either component. The encapsulation of EGCG significantly enhanced its aqueous stability and radical scavenging potential underscoring the synergistic effect of NP-mediated delivery on polyphenol bioactivity. *In vitro* investigations using THP-1-derived macrophages revealed optimal downregulation of inflammatory markers, including TNF-α and IL-8, alongside reduced NF-κB nuclear translocation. These data strongly support the hypothesis that BSA-NPs enhanced the anti-inflammatory action of EGCG by enhancing cellular uptake and sustained intracellular release, probably through endocytic pathways and controlled desorption kinetics. Considering their biocompatibility, tunable physicochemical parameters, and pronounced anti-inflammatory effects, EGCG@BSA-NPs represented a promising nanoplatform for the treatment of chronic inflammatory disorders. Future work will focus on elucidating the intracellular trafficking routes and performing *in vivo* biodistribution and pharmacodynamic assessments to fully exploit their therapeutic potential in translational settings.

## Data Availability

The raw data supporting the conclusions of this article will be made available by the authors, without undue reservation.
